# AGING APPEARANCE AND ITS SIGNIFICANCE TO WELL-BEING

**DOI:** 10.1093/geroni/igz038.2254

**Published:** 2019-11-08

**Authors:** Naomi Woodspring

**Affiliations:** University of the West of England, Bristol, United Kingdom

## Abstract

Ageing is a profoundly embodied process, yet elder’s concerns about appearance are perceived, by many, as trivial. Notions of appearance as a core human concern continues as a significant aspect throughout our lives. Self-presentation choices convey a sense of our identity. This paper is based on a qualitative study which aimed to explore current notions of beauty and age. A diverse group of postwar women (born between 1945 -1955) from the US and the UK were interviewed with a focus on their own self-presentation and the acts of seeing and being seen. This paper explores the some of the findings from this study. The majority of women, and the all women of colour, reported feeling more confident in their appearance and appreciative of other older women’s appearance. This led to a more robust sense of well-being and suggests that age and appearance may be significantly linked to well-being.

